# Post-mortem correlates of Virchow-Robin spaces detected on *in vivo* MRI

**DOI:** 10.1177/0271678X211067455

**Published:** 2022-05-17

**Authors:** Lukas Haider, Simon Hametner, Verena Endmayr, Stephanie Mangesius, Andrea Eppensteiner, Josa M Frischer, Juan Eugenio Iglesias, Frederik Barkhof, Gregor Kasprian

**Affiliations:** 1 NMR Research Unit, Queen Square Multiple Sclerosis Centre, University College London Institute of Neurology, London, UK; 2 Department of Biomedical Imaging and Image Guided Therapy, Medical University of Vienna, Wien, Austria; 3 Clinical Institute of Neurology, Centre for Brain Research, Medical University of Vienna, Wien, Austria; 4 Department of Neuroradiology, Medical University of Innsbruck, Innsbruck, Austria; 5 Department of Neurosurgery, Medical University Vienna, Wien, Austria; 6 Centre for Medical Image Computing (CMIC), Department of Medical Physics and Biomedical Engineering, University College London, London, UK; 7 National Institute for Health Research (NIHR) University College London Hospitals (UCLH) Biomedical Research Centre, London, UK; 8 Department of Radiology and Nuclear Medicine, VU University Medical Centre, Amsterdam, Netherlands

**Keywords:** Glymphatic system, histology, in vivo MRI, post-mortem MRI, Virchow-Robin spaces

## Abstract

The purpose of our study is to quantify the extent to which Virchow-Robin spaces (VRS) detected on in vivo MRI are reproducible by post-mortem MRI.

Double Echo Steady State 3T MRIs were acquired post-mortem in 49 double- and 32 single-hemispheric formalin-fixed brain sections from 12 patients, who underwent conventional diagnostic 1.5 or 3T MRI in median 22 days prior to death (25% to 75%: 12 to 134 days). The overlap of in vivo and post-mortem VRS segmentations was determined accounting for potential confounding factors.

The reproducibility of VRS found on in vivo MRI by post-mortem MRI, in the supratentorial white matter was in median 80% (25% to 75%: 60 to 100). A lower reproducibility was present in the basal ganglia, with a median of 47% (25% to 75%: 30 to 50).

VRS segmentations were histologically confirmed in one double hemispheric section.

Overall, the majority of VRS found on in vivo MRI was stable throughout death and formalin fixation, emphasizing the translational potential of post-mortem VRS studies.

## Introduction

Virchow-Robin spaces (VRS) are the anatomical spaces between the basement membrane of the perivascular astrocytic feet processes (glia limitans perivascularis) and the basement membranes of the blood vessel endothelia.^1^

Within the VRS, CSF flows from the subarachnoid space into the brain parenchyma, allowing for exchange with brain interstitial fluid (ISF). Additionally, VRS are involved in the intramural, perivascular connective tissue drainage pathway, from the brain, towards the subarachnoid space.^2^

They are thus involved in central nervous system (CNS) debris removal and lymphatic immune gateways.^3^

In healthy control subjects, the size of VRS is below the resolution of current conventional brain MRI scans at 3T.^4^ However, large Virchow-Robin spaces (VRS) are a frequent imaging finding in routine clinical MRI scans of the brain.^5^ Enlargement of the perivascular spaces has been suggested as a consequence of debris accumulation,^6^ e.g., in the course of mucopolysaccharidoses^7^ or during collagen deposition in atherosclerosis,^8^ but no singular mechanism has emerged as yet. In multiple sclerosis, a disease with chronic inflammatory and neurodegenerative components,^9^ inflammation,^10^ in line with community-based studies of ageing,^11^ as well as dilatation associated with chronic brain parenchymal atrophy,^12^ were associated with enlarged VRS^13^ and perivascular collagen deposition.^14^

Large VRS are associated with an increased risk for lacunar stroke,^15^ stroke recurrence,^16^ vascular-related cognitive impairment,^17^ oral anticoagulation-related symptomatic cerebral hemorrhage,^18^ and progression of disability in Parkinson’s disease,^19^ as well as both present and future cognitive impairment.^20–23^

Detailed histological and molecular analysis of large VRS on post-mortem tissue could further guide our understanding of CNS waste clearance and the influence of vascular pathology on CNS disorders. However, the actual histopathological correlates of VRS seen on *in vivo* MRI has remained largely unspecified, as it is still unclear at which rate large VRS were preserved after death and post-mortem tissue processing.

The purpose of our study therefore was to quantify the extent to which VRS detected on in vivo MRI are reproducible by post-mortem MRI in 12 individuals who underwent MRI at a median of 22 days before their death and in whom the brain was available for post-mortem processing.

## Materials and methods

### Study cohort

Individuals were recruited based on the following criteria: brain MRI within <one year before death; in vivo MRI with sufficient image quality to visualize and segment the VRS; and availability of post-mortem brain tissue from the same patient at our local neuropathology department. This inclusion criterion was met by twelve consecutive patients. Clinical details, including the number of analysed tissue blocks per patient, main clinical and neuropathological diagnoses, the cause of death, the time intervals from in vivo MRI to death, and from death to post-mortem MRI, are summarized in Table 1.

**Table 1.** Clinical core characteristics.

ID	Sex	Age at death [years]	MRI-Death [d]	Death -MRI [d]	N tissue blocks	Main clinical diagnosis	Cause of death	Main neuropathological findings
	
#1-19	Female	78	25	402	SH: 0DH: 5	Brain tumor; Diabetes mellitus typ II; Atherosclerosis, Gastrointestinal haemorrhages	Fever, peripheral septic conditions, hypotension	Glioblastoma, IDH1 wild type
#22-19	Female	39	241	329	SH: 6DH: 0	Decompensated hepatic cirrhosis; Hypertrophic obstructive cardiomyopathy; Chronic kidney disease	Cardiopulmonary resuscitation	Diffuse white matter injury
#42-19	Female	35	1	280	SH: 8DH: 4	Morbus Hodgkin; HIV (CDC: 3); HCV	Morbus Hodgkin with brainstem involvement	Meningeosis leucaemica, leukoencephalopathy in the right temporal lobe, prominent brain edema
#66-18	Female	57	132	618	SH: 0DH: 3	Tuberculosis; Constrictive pericarditis; Diabetes mellitus typ II	Cerebral tuberculosis	St.p. brain biopsy of right-postcentral granulomatous-necrotizing inflammation with post-vasculitic vessel abnormalities
#95-19	Male	74	135	181	SH: 4DH: 6	Diffuse large B-cell lymphoma; Sarcopenia; Acute kidney injury	Not further specified, death occurred at a palliative care unit	Meningeosis lymphomatosa, cerebral amyloid angiopathy (Thal type 2) without hemorrhagic complications
#100-19	Male	75	11	174	SH: 2DH: 7	Amyloid angiopathy; Diffuse large B-cell lymphoma; Atrial fibrillation; Arterial hypertension	Right-heart failure	Moderate meningeosis lymphomatosa
#104-19	Male	57	8	192	SH: 0DH: 8	Breast Cancer	Meningeal carcinomatosis, multiple tumor-thromboses	Breast cancer with widespread dural metastases, partial sinus thrombosis, multiple leptomeningeal vessel obliterations, multiple acute cortical microinfarcts
#112-18	Female	49	15	513	SH: 2DH: 2	Gastric cancer, Sarcopenia	Brain metastasis	Cortical infarcts left occipital, in watershed distribution, marked brain edema
#120-19	Female	77	138	123	SH: 7DH: 2	Cardiomyopathy not further specified; Chronic kidney disease; Paroxysmal atrial fibrillation; Dementia not further specified; Hypothyreosis	Hemorrhage during thrombectomy	Vessel wall amyloidosis in choroid plexus and dural vessels, partially recanalized thrombosis of the left ACM, associated subacute cortical infarct in the left temporoparietal region, moderate AD pathology, fibrotic vessel walls of small intracerebral arteries, chronic subdural hematoma
#125-19	Female	67	17	104	SH: 3DH: 5	Recurrent craniopharyngioma	Cardiorespiratory failure	St.p. adamantinomatous craniopharyngeoma resection, severe ischaemic cerebellar Purkinje cell loss, diffuse arteriolosclerosis
#140-19	Female	52	36	103	SH: 0DH: 4	Adenocarcinoma of the lacrimal gland	Multi organ failure	St.p. opticus glioma resection, St.p. resection of carcinoma of lacrimal gland, marked diffuse brain edema, cystic tissue defects in both basal rostral basal ganglia (diameter 2.5 cm), Dura with tumour infiltration in the region of the tractus opticus
#151-18	Female	77	19	409	SH: 0DH: 3	Diffuse large B-cell lymphoma; Venous thromboembolism; Acute kidney injury	Multi organ failure under peripheral septic conditions	Moderate brain edema, mild unspecific terminal brainstem encephalitis, iron deposition and axonal degeneration in the basal ganglia reminiscent of neurodegeneration with brain iron accumulation
	Female: male = 9:3	median: 62 (25% to 75%: 50 to 77)	median: 22 (25% to 75%: 12 to 134)	median: 236 (25% to 75%: 136 to 407)	Total: SH: 32DH: 49			

Note: Sex, age, the time interval from last *in vivo* MRI to death, the time interval from death to the post-mortem MRI, and clinical core diagnosis/cause of death are summarized for each study subject.

SH: single-hemispheric; DH: double-hemispheric.

The tissue slicing was performed in the course of neuropathological clinical routine at standardized section levels, including coronal sections at the frontal poles, at the level of the temporal poles, a mid-thalamic section level and occipital sectioning. The availability of these tissue sections for this study was determined by prior neuropathological diagnostic work-up, regions of clinical interest, typically those including lesions, were utilized and not available for our subsequent analysis. The entire available brain tissue was analyzed in each patient. In total we investigated 81 tissue-blocks, of which 49 were double- and 32 single-hemispheric/lobar formalin-fixed brain sections; On a patient basis in median 4 double-hemispheric sections (25%–75%: 3–5), and 2 single-hemispheric sections were included (25%–75%: 0–5) Table 1.

### MRI acquisition in vivo

*In vivo* MRI acquisition was performed in the course of the clinical routine, with imaging adapted to the clinical question and thus not standardized across all patients. However, the imaging protocols were quite similar, as all patients were imaged in our department. A 1.5 Tesla Philips Ingenia MRI with an eight-channel head coil was used in 10/12 subjects. The remaining two subjects were scanned with a Philips Achieva (3T, 32-channel head coil) and a Siemens Vida (3T, 64-channel head coil), respectively. 3D-T1-weighted images were available in all cases. A detailed list of the analyzed *in vivo* MRI sequences is available in Supplementary Table 1.

### MRI acquisition post-mortem

From the included 12 subjects, a total of 49 double- and 32 single-hemispheric/lobar formalin-fixed brain sections, with a slice thickness of around 1 cm were obtained for the post-mortem MRI analysis, the sections were cut prior to formalin fixation. Tissue sections were axially oriented in one subject (#22-19), which was anaylsed axially, wherease coronal reformates were used in all the other cases. Post-mortem scanning was performed with a Siemens Vida 3T scanner.

Formalin-fixed tissue blocks were individually placed in a flat plastic container and fully emerged in Flourinert.^24^

Double Echo Steady State (DESS) images were acquired using the built-in spine coil of the table, which has 72 channels in 12 segments, of which we used four, and the Siemens “multiflex surface coil,” with 18 channels on top of the specimen.

T2-weighted DESS images were acquired with an echo time of 5.6 ms, a repetition time of 17 ms, and a flip angle to 25°. The isotropic resolution was 0.4 mm (matrix: 468 × 576), and the acquisition time, per tissue block, was around 30 to 50 min. Due to the immersion in Flourinert the brain is surrounded by extremely low signal intensity despite the T2-weighted contrast.

### Histology acquisition

To test if VRS segmented on MRI correspond to VRS histologically, proof of concept was tested in one double hemispheric section from ID #120-19. The section was stained with Hematoxylin Eosin, Elastica van Gieson, and Luxol fast-blue myelin stain as reported previously.^25^ Histological images were manually co-registered to the aligned post-mortem and in-vivo MRI images.

### Image analysis

In a first step, VRS were manually segmented with 3D-Slicer^26^ on the *in vivo* data set (AE, SM, LH) and subsequently on the post-mortem set (AE, SM, LH) in the entire brain. Lesions were masked from the analysis (LH). The *in vivo* segmentation were performed without knowledge of the post-mortem images. As suggested previously (STRIVE-criteria), VRS were defined as CSF iso-intense lines following the course of vessels in the supratentorial white matter and deep gray matter.^1^

The post-mortem MRIs were manually and rigidly co-registered with the corresponding *in vivo* MRIs based on anatomical landmarks with Freeview/FreeSurfer.^27^ VRS segmentations were not used as additional information/landmarks for the registration process.

**Figure 1.**
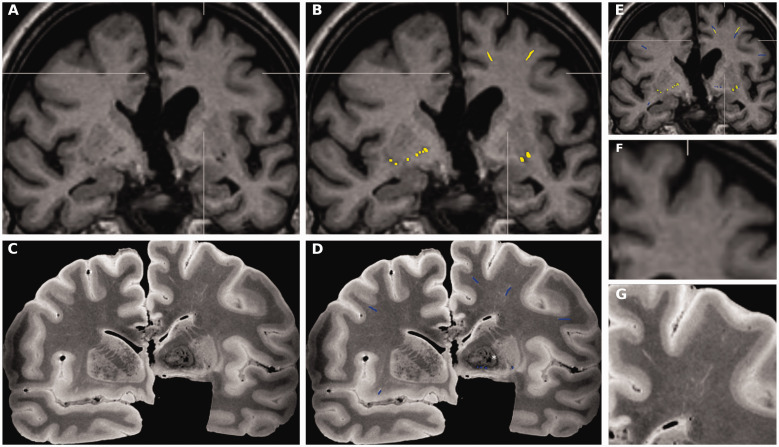
VRS *in vivo* vs. post-mortem quantification. All images in Figure 1 are taken from a female subject (#125-19), 67-years of age, who died 17 days after the MRI acquisition (see Table 1 for details). (a) On *in vivo*T1-weighted coronal images, two VRS are depicted in the medial/superior frontal gyrus and middle frontal gyrus in the left hemisphere (detailed in insert f) and bilaterally at the level of the perforating lenticulostriatal arteries. (b) VRS were manually segmented on *in vivo* images in the entire brain without knowledge of the post-mortem images (yellow color). (c) On T2-weighted images from the corresponding post-mortem sections, additional VRS are visualized in the right middle frontal gyrus (detailed in insert g), right insula, and left inferior frontal gyrus. (d) VRS were manually segmented on the entire T2 weighted images, without knowledge of the *in vivo* images. (c and d) Areas of low signal intensity are noted in the left globus pallidum (in the right side of the image, indicated with *), findings are consistent with idiopathic calcium depositions, a frequent incidental finding in elderly subjects. (e) After manual registration/alignment of post-mortem with *in vivo* images, VRS segmentations from both sessions were displayed on top of the *in vivo* scan and the number of *in vivo* VRS with the corresponding post-mortem VRS were determined at the section level with the highest anatomical alignment. (f) *In vivo* T1-weighted coronal images, magnified from (a). (g) Post-mortem T2-weighted coronal images, magnified from (c). VRS: Virchow-Robin Spaces.

The amount of overlap of the *in vivo* and the post-mortem VRS segmentations were manually quantified for each tissue-block using Freeview/FreeSurfer by LH/SM. To account for the inherent tissue distortion that occurs between *in vivo* and post-mortem imaging and the limited slice thickness (∼1cm), the analysis was limited to one section level within each tissue-block with the highest spatial correspondence of anatomical landmarks. VRS were counted on *in vivo* MRI scans with both segmentations from *in vivo* and post-mortem MRI overlaid at 50% opacity in Freeview. We collected the counts of VRS where the segmentations overlapped or did not overlap for each tissue block (Figure 1).

### Statistical analysis

The statistical analysis was calculated with R-studio.^28^

The ratio of *in vivo* VRS with and without post-mortem correlates was collected on a tissue-block basis and averaged per patient for two anatomical areas: deep gray matter (including thalamus or putamen and globus pallidum) and supratentorial white matter. The rates are provided with medians and a 25% to 75% range.

In tissue blocks with no VRS on either *in vivo* or post-mortem MRI, which was the case in 4/20 blocks with deep gray matter, we did not calculate or assign a detection rate.

To account for the potential effect of confounders on the post-mortem detection rate of VRS as imaged on in vivo MRI, we used linear mixed-effect regression models. These models were separately calculated for supratentorial white matter and deep gray matter. Fixed-effect variables included in these two models were the *in vivo* MRI-to-death interval [days], the death-to-post-mortem MRI interval [days], whether tissue-blocks were single-hemispheric/lobar or double-hemispheric, and the surplus of VRS found on post-mortem MRI relative to the total number of VRS in this tissue block, ie., [(post-mortem – *in vivo*)/(post-mortem + *in vivo*)] and the total number of tissue blocks analyzed per patient . These models used the individual tissue block as the unit of analysis. To account for the nested structure of the data, tissue blocks were nested within patients. We report the beta for each predictor with its 95% confidence interval and p-value.

Normality was visually assessed with quantile-quantile- and density plots and qualitatively with the Shapiro-Wilk test.

Inter-rater agreement was assessed with inter-class correlation coefficients, which are provided with their respective 95% confidence interval and p-value.

### Data availability statement

Anonymized data, not published in the article, will be shared upon reasonable request from a qualified investigator after approval from the local Ethics committee.

The study was approved by the ethics committee of the Medical University Vienna and performed in accordance with the Helsinki Declaration. Written, informed consent was obtained from all subjects or their legal representatives as far as required by Austrian law.

## Results

### Cohort characteristics

Twelve subjects contributed a total of 49 double- and 32 single-hemispheric/lobar formalin-fixed brain sections. The median age at death was 62 years (25% to 75%: 50 to 77-years). The time from MRI to death was a median of 22 days (25% to 75%: 12 to 134 days). The formalin fixation time at post-mortem MRI acquisition was a median of 236 days (25% to 75%: 136 to 407 days). Subjects (female: male = 3: 9) died during the course of various conditions, including malignant/metastatic disease (n = 8), decompensated hepatic cirrhosis with cardio-pulmonary resuscitation (n = 1), cerebral vascular occlusion (n = 1), constrictive pericarditis and cerebral tuberculosis (n = 1), and amyloid angiopathy (n = 1) (see Table 1 for details). Supratentorial white matter was present in all tissue blocks (n = 81) and thus in all subjects, the deep gray matter was present in 20/81 tissue blocks and absent in three subjects.

#### Detection rates of in vivo MRI VRS by post-mortem MRI

Supratentorial white matter was present in all tissue blocks (n = 81) and thus in all subjects.

On a patient basis, a median of 80% (25% to 75%: 60 to 100) of VRS found on *in vivo* MRI were reproduced at post-mortem imaging in the supratentorial white matter, which was in absolute numbers a median of 15 VRS/patient (25% to 75%: 9 to 36).

The deep gray matter was present in 20/81 tissue blocks and absent in three subjects.

**Figure 2.**
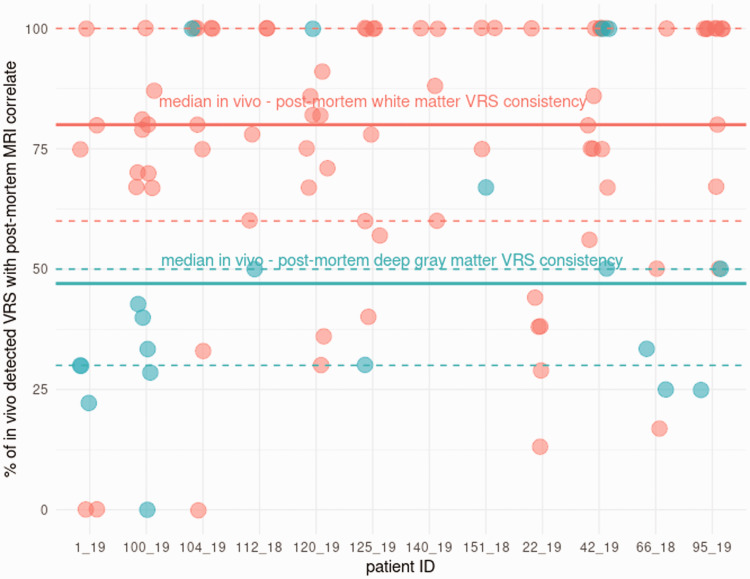
*In vivo* – post-mortem VRS consistency. The *in vivo* - post-mortem MRI VRS consistency is provided for each tissue-block, grouped per patient and the location in the white matter or the deep gray matter (white matter = red; deep gray matter = blue). Median (thick line) and 25% to 75% range (dashed lines) were calculated for white matter and deep gray matter. VRS: Virchow-Robin spaces.

Overall, the percent of *in vivo* VRS reproduced on post-mortem images was lower in the deep gray matter than in the white matter, with a median of 47% (25% to 75%: 30 to 50), and a median of 1.5 VRS/patient (25% to 75%: 0 to 5.8) (Figure 2).

### Influence of confounding factors on the detection rate of in vivo MRI VRS by post-mortem MRI

To assess the potential influence of confounders on the *in vivo* - post-mortem VRS consistency, nested linear mixed-effect regression models were computed separately for white matter and deep gray matter (Table 2).

**Table 2.** Influence of potential confounders on the rate of VRS detected on both *in vivo* and post-mortem MRI.

	Deep gray matter model			White matter model		
Predictors	Est.(β1)	CI (95%)	p	Est.(β1)	CI (95%)	p
Intercept	98.45	–81.27–278.17	0.242	67.30	29.66–104.94	**0.001**
Single hemispheric block	53.73	23.34–84.12	**0.004**	1.40	–12.40–15.20	0.840
n VRS (p.m.−i.v.)/(p.m.+i.v.)	–14.27	–30.85–2.31	0.082	14.07	–1.72–29.86	0.080
Time: MRI to death [d]	0.04	–0.48–0.56	0.854	–0.08	–0.20–0.04	0.165
Time: death to MRI [d]	–0.10	–0.38–0.19	0.440	–0.01	–0.08–0.06	0.728
Total number of blocks/patient	–2.99	–18.97–12.98	0.663	1.46	–2.23–5.14	0.389

Note: Linear mixed effect models, with tissue block nested in subjects as a random factor, were computed for the following confounders: total number of VRS *in vivo*, total number of VRS post-mortem, the time interval from *in vivo* MRI to death, and the time interval from death to the post-mortem MRI. None of the confounders had a significant effect size.

MRI: magnetic resonance imaging; VRS: Virchow-Robin spaces.

On an individual block basis (supratentorial white matter n = 81, deep gray matter n = 20), there were no effects of the time-interval from *in vivo* MRI to death or the time-interval from death to post-mortem MRI on the *in vivo* - post-mortem VRS consistency.

Similarly, in both locations, the surplus of VRS found on post-mortem images, relative to the total number of VRS in this block, was not related to the *in vivo* - post-mortem VRS consistency rate.

In the deep gray matter, however, single-hemispheric or lobar tissue-blocks were associated with higher *in vivo* - post-mortem VRS consistency than deep gray matter regions from double-hemispheric blocks (beta = 52, 95%CI: 23–82, p = 0.004) (Table 2).

The total number of tissue-blocks analyzed per patient was not related to the *in vivo* - post-mortem VRS consistency (deep gray matter model: p < 0.663; white matter model: p < 0.389).

The inter-rater agreement on VRS within in-vivo MRIs was 0.91 (95% CI: 0.86–0.95, p < 0.001) and in the post-mortem MRIs: 0.92 (95% CI: 0.87–0.96, p < 0.001).

**Figure 3.**
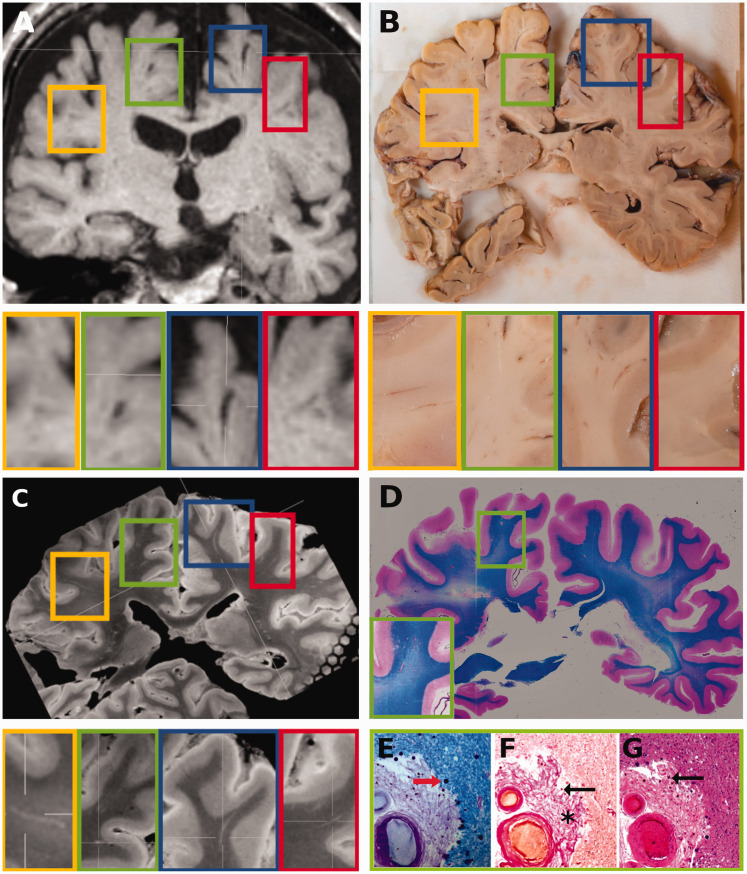
VRS- *in vivo* MRI – post-mortem MRI – histology. All images in Figure 3 are taken from the same subject (#120-19), a female who died at 77 years of age after middle cerebral artery occlusion and hemorrhage during thrombectomy. (a) 3 D-T1-weighted *in vivo* MRI at 1.5 T acquired 138 days prior to death revealed ∼six large VRS at the given mid-thalamic imaging plane in the frontal deep white matter. Four VRS are indicated with colored rectangles and shown in corresponding higher resolution images below. (b) Some VRS (e.g., in the yellow and blue rectangle), but not all VRS (e.g., green and red rectangle) are seen macroscopically on the surface of the corresponding tissue block. (c) The *in vivo* MRI-detected VRS are visible on post-mortem MRI at the same level in the yellow, green, blue, and red rectangles. (d) Coronal double-hemispheric Luxol fast blue-stained section at the same level shows a large VRS in the right superior/medial frontal gyrus that was also visible on the *in vivo* and post-mortem MRI (indicated by the green rectangle and the higher magnification of this area in the green insert in the left lower corner of the image). (e) A higher magnification image of the Luxol fast blue staining reveals perivascular corpora amylacea (red arrow) and abundant connective-tissue in the VRS. (f) Corresponding Elastica van Gieson staining shows hyalinized vessel walls and extensive perivascular collagen deposition (asterisk), filling and dilating the in the perivascular space. Additionally small hemosiderin depositions are noted (black arrow). (g) Corresponding Hematoxylin Eosin staining shows thickening of the adventitia and media hyalinosis with some hemosiderin depositions (black arrow) in this perforating artery in the deep white matter. Only slight perivascular edema and tissue rarefaction is evident. VRS: Virchow-Robin spaces,

In three tissue blocks, the *in vivo* – post-mortem VRS reproducibility was 0% (Figure 3). Three of those blocks included white matter (ID 1-19, block 1, -2; ID 104-19, block 10), and one included deep gray matter (ID 100-19, block 3). In all four blocks, only a small number of short and thin *in vivo* VRS were present (*in vivo* VRS count = 1–2). The post-mortem MRI of these blocks showed similarly low VRS counts (post-mortem VRS count ID 1-19-block 1/2: 3, 104-19 block 10: 6, ID 100-19 block 3: 1).

### Histological validation

A histological proof-of-concept was achieved in one double hemispheric section from subject #120-19, (Figure 3).

Numerous VRS were perceivable on *in vivo* MRI and post-mortem MRI (Figure 3) of which one VRS around a perforating artery in the deep white matter of the right superior/medial frontal gyrus was present in the histological section level (Figure 3). Elastica van Gieson staining shows media hyalinosis and extensive perivascular collagen deposition with filling with expansion of the perivascular space. The adventitia is thickened and the surrounding tissue shows slight perivascular tissue rarefaction.

**Figure 4.**
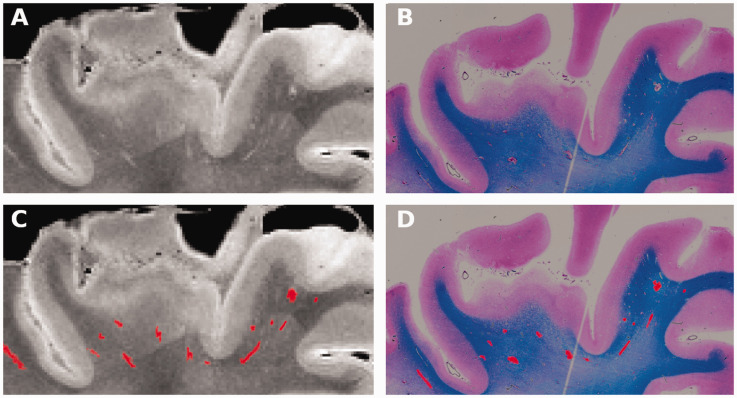
VRS – post-mortem MRI – histology. (a) Post-mortem MRI (from subject #120-19) showing a high magnification view of the right superior frontal gyrus. VRS are seen as bright lines in the white matter beneath the cortex. (b) Corresponding VRS are seen in a co-registred luxol fast blue-stained histological section at the same level. (c and d) VRS segmentations are highlighted in red on post-mortem MRI and histology. VRS: Virchow-Robin spaces.

Comparing post-mortem MRI (Figure 4(a), right superior frontal gyrs from subject 120-19) with corresponding histological images (Figure 4(b), luxol fast blue staining) a close spatial correlation between the segmented VRS on MRI and histology is observed (Figure 4(c) and (d)) suggesting correct visualization of VRS by post-mortem MRI.

## Discussion

Virchow-Robin spaces (VRS) are increasingly recognized to be involved in the pathogenesis of various neurological conditions. While VRS are readily visualized on *in vivo* MRI, the histo- biological correlates of those VRS seen on *in vivo* MRI remains uncertain.

Comparability between *in vivo* imaging and post-mortem analysis is a necessary prerequisite for further investigation of the histological and biological basis of pathologies that originate in or involve this specific brain compartment. In the present cohort we therefore tested the hypothesis if VRS detected on in vivo MRI are reproduced by post-mortem MRI. In 12 subjects with 81 tissue blocks, the majority of VRS detected on in vivo MRI were reproducible at post-mortem imaging, providing the first quantification of the translational potential of post-mortem VRS studies.

Large VRS have been associated, among other conditions, with stroke,^15,16^ hemorrhage,^18^ and cognitive impairment.^17,19–23,29^ Further understanding of the histological and biological processes that occur within large perivascular spaces could thus facilitate our understanding of relevant disease processes. Their enlargement on *in vivo* MRI has been suggested to occur as a consequence of debris accumulation,^6,29^ but VRS are also considered to be fluid-filled spaces^1^ and perivascular collagen depositions have been reported in the perivascular space.^8,14^ While the histological validation in our study was limited to 1/12 cases, we found that perivascular collagen depositions were associated with VRS that remained visible from *in vivo* imaging to post-mortem imaging.

Within the supratentorial white matter, where VRS are most abundant,^30^ 80% of VRS seen on *in vivo* MRI were also observed on post-mortem images. This is a high detection rate considering the underlying variability (see Limitations section). In the deep gray matter, the rate of the VRS seen on *in vivo* MRI post-mortem was 47%. The lower reproducibility in the deep gray matter could be due to the smaller sample size of this structure and inherent technical reasons, e.g., the registration technique, manual alignment of anatomical landmarks, could be more efficient with gyri and sulci than with deep gray matter structures, and thus, favor alignment of supratentorial white matter. The lower reproducibility of deep gray matter VRS could also reflect biological differences in the VRS. Histologically, VRS in the deep gray matter are distinct from those in the cortex/white matter, as they are surrounded by two distinct layers of leptomeninges separated by a perivascular space that is continuous with the subarachnoid space.^31^ They are also clinically distinct, as large VRS located around the perforating lenticulostriate arteries are frequently observed due to their developmental origin, associated with glial tunnels during axonal growth of the forming anterior commissure.^32^

This study has several limitations. Although VRS were rated within the same patient *in viv*o and post-mortem, the interval from *in vivo* MRI-to-death was short (a median of 22 days), and, although post-mortem images were carefully aligned with *in vivo* MRI (Figures 1 and 3), alignment is never perfect. This has several reasons and major implications for the interpretation of our results. In the present cohort, brain pathology was directly related to morbidity. Thus, even in a median 22-day interval from *in vivo* MRI to death, some of the *in vivo* dynamics in VRS remain uncertain. However, we did not observe significant effects of the in vivo MRI to death- and the death to post-mortem MRI time-intervals or other potential confounders, such as the surplus of VRS found on post-mortem images on the overall in vivo - post-mortem VRS consistency rate, using nested linear mixed-effect regression models. Furthermore, the brain is distorted in the course of tissue processing and fixation, with around 8% global volume shrinkage over a 70-day fixation period.^33^ Further, when fresh brain is cut into slices, the section level is not strictly within the coronal plane that was defined by *in vivo* MRI. To some extent, this can be corrected with careful manual alignment guided by anatomical landmarks, such as gyri and sulci in 3D data sets, as available in our study. To further account for tissue distortion, the analysis was limited to one section level within the tissue block with the highest anatomical correspondence with *in vivo* images. Given the post-mortem slice thickness of around 1 cm and the longitudinal course of VRS, we could thus achieve a representative quantification for each block, while preventing over-fitting. We further tried to avoid over-fitting by applying a registration that allowed movement within the three spatial directions and adjustment of the overall size of the post-mortem block, but we did not allow local distortion/spatial transformations. Thus, as also visually evident from Figures 1 and 3, we can only provide a rough estimate for the rate at which *in vivo*-defined VRS could be found post-mortem.

Another limitation of our study is the sample size (12 cases within a total of 49 double- and 32 single-hemispheric/lobar tissue blocks) with its inherent statistical limitations. In order to maximize the available tissue, we thus included all sections available, leading to differences in the total number of analysed tissue-sections across subjects. In median 4 double-hemispheric sections (25%–75%: 3–5) and 2 single-hemispheric sections were included (25%–75%: 0–5). However, the total number of sections analysed per patient did not show significant effects on the *in vivo* - post-mortem VRS consistency rates.

The biological processes that lead to the enlargement of VRS will likely differ, e.g., active perivascular inflammation vs. atrophy vs. perivascular collagenosis, etc., and we can not account for this heterogeneity in this cohort.

We observed higher counts of VRS post-mortem than *in vivo* (Figures 1 and 3), which is not surprising given the higher resolution applied post-mortem (0.4 mm vs. 1 mm isotropic voxel size, 3T vs. mainly 1.5T *in vivo*) and overall inter-rater agreement was high (>90% both in vivo and post-mortem). However, we can neither exclude artificial “VRS opening” in the course of tissue fixation or ascertain the rate of VRS that may have evolved between *in vivo* MRI and death. While the scanners were theoretically available to scan the blocks on the same machine on which the *in vivo* images were acquired, the nature of formalin tissue fixation does not allow acquisition of the identical sequences used *in vivo* and thus hinder a 1:1 comparison.^34^

The proof of concept, that the VRS segmented on MRI correspond to enlarged perivascular spaces histologically was performed in on patient (#120-19), Figures 3 and 4. We observed a close correlation between histologically confirmed VRS and post-mortem MRI segmentations in this subject (Figure 4(c) and (d)). While depositions of collagen, filling and expanding the perivascular space, were observed in this subject (asterisk in Figure 3(f)) that might explain the persistency of these spaces across time and during tissue fixation, we can not extrapolate this observation to other individuals. In line we did not investigate histological factors that influence VRS in vivo vs. post-mortem persistency rates and it was unfortunately not possible for us to ascertain if perivascular space enlargement was peri-arterial or peri-venous. Case 120-19 was chosen because death was related to an unexpected sudden event (haemorrhage during thrombectomy), rather than a progressive disease process, and because of the overall high tissue and MRI quality.

The high number of VRS on post-mortem scans could arguably also increase the *in vivo* post-mortem detection rate via “false-positive VRS.”, however this theory is not supported by our data, since, on a block basis, the total number of VRS, neither *in vivo* or post-mortem, were not predictive for the *in vivo* post-mortem VRS detection rate.

## Conclusion

In conclusion, the majority of VRS found on in vivo MRI, was observed on post-mortem imaging, thus suggesting that histological and biochemical analysis of VRS detected by post-mortem MRI could guide future research in this evolving field.

## Supplemental Material

sj-xlsx-1-jcb-10.1177_0271678X211067455 - Supplemental material for Post-mortem correlates of Virchow-Robin spaces detected on *in vivo* MRI Click here for additional data file. Supplemental material, sj-xlsx-1-jcb-10.1177_0271678X211067455 for Post-mortem correlates of Virchow-Robin spaces detected on *in vivo* MRI by Lukas Haider, Simon Hametner, Verena Endmayr, Stephanie Mangesius, Andrea Eppensteiner, Josa M Frischer, Juan Eugenio Iglesias, Frederik Barkhof and Gregor Kasprian in Journal of Cerebral Blood Flow & Metabolism
